# The immediate and long-term effects of exercise and patient education on physical, functional, and quality-of-life outcome measures after single-level lumbar microdiscectomy: a randomized controlled trial protocol

**DOI:** 10.1186/1471-2474-7-70

**Published:** 2006-08-25

**Authors:** David M Selkowitz, Kornelia Kulig, Elizabeth M Poppert, Sean P Flanagan, Ndidiamaka D Matthews, George J Beneck, John M Popovich, Jose R Lona, Kimiko A Yamada, Wendy S Burke, Carolyn Ervin, Christopher M Powers, Physical Therapy Clinical Research Network (PTClinResNet)

**Affiliations:** 1 Department of Biokinesiology and Physical Therapy, University of Southern California, 1540 East Alcazar St., Los Angeles, CA 90089, USA; 2 Division of Biostatistics & Epidemiology, Keck School of Medicine, University of Southern California, 1540 Alcazar St., CHP 222, MC 9010, Los Angeles, CA 90033, USA; 3 Department of Physical Therapy Education, Western University of Health Sciences, 309 E. Second St., Pomona, CA 91766, USA; 4 Department of Kinesiology, California State University at Northridge, 18111 Nordhoff St., Northridge, CA 91330, USA; 5 Department of Physical Therapy, California State University, Long Beach, Long Beach, CA 90840, USA

## Abstract

**Background:**

Low back pain remains a costly quality-of-life-related health problem. Microdiscectomy is often the surgical procedure of choice for a symptomatic, single-level, lumbar disc herniation in younger and middle-aged adults. The question of whether a post-microdiscectomy exercise program enhances function, quality of life, and disability status has not been systematically explored. Thus, the overall purpose of this study is to assess immediate and long-term outcomes of an exercise program, developed at University of Southern California (USC), targeting the trunk and lower extremities (USC Spine Exercise Program) for persons who have undergone a single-level microdiscectomy for the first time.

**Methods/design:**

One hundred individuals between the ages of 18 and 60 who consent to undergo lumbar microdiscectomy will be recruited to participate in this study. Subjects will be randomly assigned to one of two groups: 1) one session of back care education, or 2) a back care education session followed by the 12-week USC Spine Exercise Program. The outcome examiners (evaluators), as well as the data managers, will be blinded to group allocation.

Education will consist of a one-hour "one-on-one" session with the intervention therapist, guided by an educational booklet specifically designed for post-microdiscectomy care. This session will occur four to six weeks after surgery. The USC Spine Exercise Program consists of two parts: back extensor strength and endurance, and mat and upright therapeutic exercises. This exercise program is goal-oriented, performance-based, and periodized. It will begin two to three days after the education session, and will occur three times a week for 12 weeks.

Primary outcome measures include the Oswestry Disability Questionnaire, Roland-Morris Disability Questionnaire, SF-36^® ^quality of life assessment, Subjective Quality of Life Scale, 50-foot Walk, Repeated Sit-to-Stand, and a modified Sorensen test. The outcome measures in the study will be assessed before and after the 12-week post-surgical intervention program. Long-term follow up assessments will occur every six months beginning one year after surgery and ending five years after surgery.

Immediate and long-term effects will be assessed using repeated measures multivariate analysis of variance (MANOVA). If significant interactions are found, one-way ANOVAs will be performed followed by post-hoc testing to determine statistically significant pairwise comparisons.

**Discussion:**

We have presented the rationale and design for a randomized controlled trial evaluating the effectiveness of a treatment regimen for people who have undergone a single-level lumbar microdiscectomy.

## Background

Approximately 12–33% of the adult work force is affected by low back pain each year, and it has been suggested that between 70% and 95% of adults will suffer from low back pain at some time during their lifetime [1-5]. Unlike other orthopedic disorders, the expenses associated with management of back pain have increased over the last 20 to 30 years [1,2]. A recent study on health care expenditures for people with back pain in the United States reported that total expenditures were over $90 billion in 1998, and that these individuals incurred 60% higher expenditures than those without back pain [6]. Despite continuous clinical and scientific efforts, low back pain remains an ever-present, quality-of-life-related, health problem.

The lumbar intervertebral disc is susceptible to injury and early degeneration, which often result in pain and disability. To effectively remedy these problems, treatment of symptomatic lumbar disc herniations has continuously evolved. Surgeons have sought to reduce operative trauma to the spine by adopting minimally invasive techniques such as microdiscectomy, (microscope-assisted or endoscopic), which is used to treat single-level disc injury in working-age adults. Despite widespread use of this technique, there are few reports of long-term results [7-10]. Early success rates ranging from 70–91% have been reported [7,11-14]. Limited available data related to long-term follow-up demonstrates that the success rate decreases to 60–70% after three to ten years [8]. However, these success rates may be influenced by post-operative care.

The current practice pattern for management of activity post-operatively has three distinct directions. A common, but not studied, approach includes 4 to 16 weeks of limited activity following the surgery. A second approach, advocated more recently by Carragee et al [15], suggests that eliminating post-operative activity restriction leads to enhanced short-term outcomes for patients after limited open discectomy. A third approach incorporates an early strengthening intervention into the post-surgical regimen [16-19].

A limited number of studies have evaluated the impact of trunk strengthening programs on functional outcomes following lumbar disc surgery. These studies varied in sample size (21–96 subjects), intervention period (4–12 weeks), and follow-up period (0–52 weeks after surgery) [16,18-21]. While the data suggest that longer interventions (three months) have a greater impact on disability status of subjects than shorter interventions [16,18,19,22], the relatively short follow-up periods make it difficult to assess the long-term effects of an exercise intervention on function, quality of life, disability status, or pain. The paucity of detail in published studies regarding the specifics of the interventions creates a challenge to researchers and clinicians attempting to reproduce the protocols.

In summary, the question of whether a post-microdiscectomy exercise program enhances long-term function, quality of life, and disability status has not been systematically explored. Thus, the overall purpose of this study is to assess immediate (after12 weeks of intervention) and long-term (up to 5 years) outcomes of a strengthening and endurance physical therapy program targeting the trunk and lower extremities for persons who have undergone a single-level microdiscectomy for the first time.

We hypothesize that compared to patient education only, a focused intervention combining muscle strengthening and endurance exercise with patient education will result in immediate and long-term improvement in selected physical measures, function, and quality of life, and will reduce pain and disability in people status-post single-level lumbar microdiscectomy.

## Methods/design

**Figure 1:**
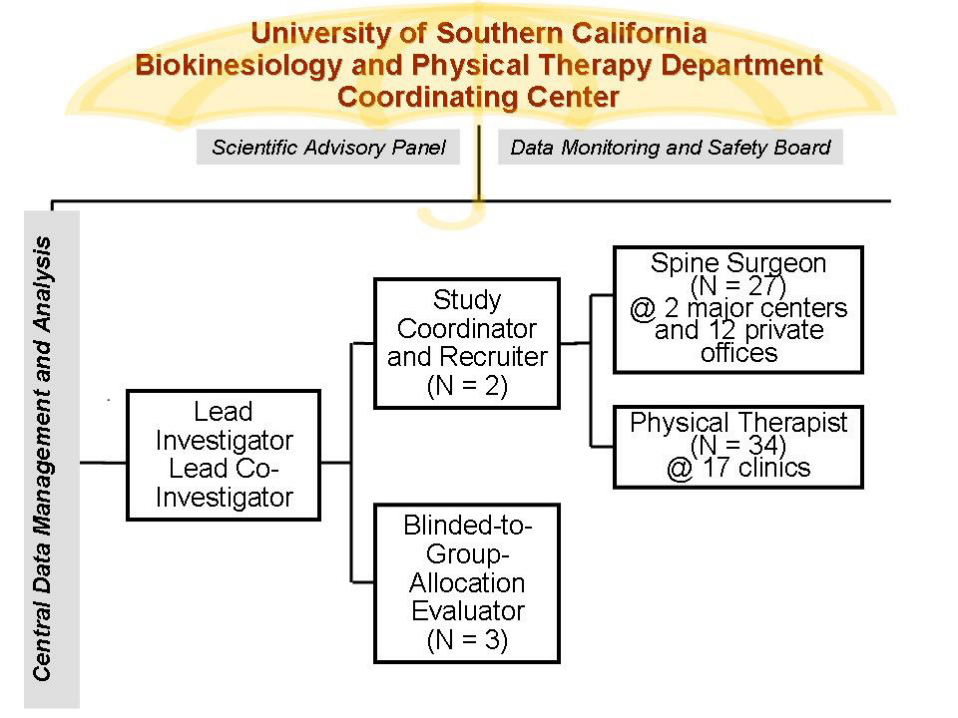
Organizational Infrastructure of the Study.

The Institutional Review Board of the University of Southern California (USC) granted approval for this randomized controlled trial. The organizational infrastructure includes a central data management and analysis team, a scientific advisory panel, and a data monitoring and safety board (Figure 1). The study coordinator and recruiter will communicate with surgical practices, physical therapy clinics, and prospective subjects. Blinded-to-group-allocation evaluators will administer all face-to-face measurements (primary and secondary outcome measures) after completing standardization training in the testing procedures. These "blinded," standardized testers will be videotaped during mock trials and their performance will be scored by other research associate examiners on their ability to administer the protocol as instructed. Standardized testers will have scored at least 90% on a checklist of competencies during mock trials before they will be permitted to test the study subjects. Physical therapists in participating clinics ("intervention therapists") will complete standardized training in the intervention procedures, including instruction of the education session and the exercise program. These intervention therapists must pass a videotaped mock intervention session with a score of 90% on a checklist of competencies before they will be permitted to administer interventions to the subjects.

The study is divided into three phases, a protocol development phase, an implementation or intervention phase, and a follow-up phase. During the protocol development phase, a team of physical therapist researchers and clinicians developed, tested, and standardized the post-surgical intervention and testing protocols. During the intervention phase, a two-group, pretest-posttest design will be used, with participants being randomly assigned to one of two groups: one session of back care education, or a back care education session followed by the 12-week USC Spine Exercise Program. This design will allow for the analysis of the effects of treatment over time. The outcome measures in the study (see below) will be assessed before and after the post-surgical intervention period. The evaluators, as well as the data managers, will be blinded to group allocation. After the end of the intervention phase, follow-up assessments of selected outcome measures will be conducted on all participants every six months, beginning one year and ending five years after the date of surgery (the follow-up phase). The follow-up phase will allow for the analysis of the stability of the effect of treatment over time.

### Study sample

One hundred individuals between the ages of 18 and 60 who consent to undergo lumbar microdiscectomy will be recruited to participate in this study. Potential subjects will be medically screened, pre- and post-surgically, by one of 27 surgeons who have been informed about the study. Patients deemed eligible for the study based on the medical screening, will be offered the opportunity by the surgeon's nurse coordinator to consult with a study coordinator regarding participation in the study. The nurse coordinator will complete an eligibility screening form on each individual screened for the study, and the study coordinator will track the number of potential participants screened and the reasons for exclusion from the study.

If the potential participant agrees to meet the responsibilities of the study, an enrollment appointment will be scheduled with a "blinded" evaluator. The evaluator will review the informed consent, and a summary of the Health Insurance Portability and Accountability Act of 1996 (HIPAA) policy, in detail with prospective participants. The purpose of the study, the procedures to be employed, any potential risks and benefits of participation, random placement into one of two groups, and the responsibilities of the participants and the members of the research team, will be discussed with the potential participants. All questions regarding the study will be answered fully. When the individual signs the consent form, the evaluator will initiate testing of the outcome measures. Successful completion of the evaluation will be reported to the study coordinator, who will inform the study participant about his or her group allocation. The study participant will choose one of the 17 participating clinics as the site of care delivery.

There will be no enrollment restrictions based on gender, race, or ethnic origin. There is no reason to expect that this study population will not reflect an adequate racial cross-section of Los Angeles and surrounding communities.

### Inclusion and exclusion criteria

The participating surgeons will screen patients for pre-surgical inclusion and exclusion criteria. The primary pre-surgical inclusion criteria include: diagnosis of disc protrusion confirmed by magnetic resonance imaging (MRI) testing, predominant symptoms in the lower extremity, radicular pain distribution, restricted straight-leg raise, and positive signs of adverse nerve-root tension (i.e., impaired mobility and/or pain and/or dysesthesia). Pre-surgical exclusion criteria include symptoms suggestive of facet arthrosis or neurogenic claudication, and plane radiographs showing more than 50 percent loss of disc height at the relevant spinal level. Patients in whom the protrusion occupies more than 50 percent of the sagittal diameter of the spinal canal or in whom sequestrated fragments are seen on MRI will also be excluded.

Individuals over the age of 60 will be excluded to control for the confounding effects of spinal osteoarthritis. Pediatric subjects will not be recruited for this study, as performing microdiscectomies in children is contraindicated.

Other pre-surgical exclusion criteria include: previous back surgeries, presence of any concurrent lower extremity pathology (other than that associated with low back and lower extremity pain associated with single level disc injury), neurological disorders (e.g., stroke, dementia, seizures), cognitive dysfunction (e.g., traumatic brain injury, cerebrovascular accident, Alzheimer's disease), uncontrolled cardiovascular disease, evidence of spinal cord compression, uncontrolled hypertension, infection, severe respiratory disease, pregnancy, rheumatic joint disease, peripheral vascular disease with sensory loss at the foot, or any condition the subject identifies that might limit participation in physical activity.

To continue in this study, the primary post-surgical inclusion criterion is that the subject must have undergone a single level (L3–4, L4–5, or L5–S1) microdiscectomy that was without adverse event four to six weeks after the surgery. All subjects must be willing to participate upon enrollment.

### Interventions

The interventional protocol comprises two components: education and exercise. The protocol is based on extensive literature review, best evidence available, and clinical expertise of those who developed the protocol, with input from consultants.

Education will consist of a one-hour "one-on-one" session with the intervention therapist, and will occur in a time window four to six weeks after surgery. Exercise will consist of two parts: 1) isometric trunk strength and endurance training, and 2) mat and upright therapeutic exercises. The exercise protocol will begin during the session following the education session (two to three days later), and will occur three times a week for 12 weeks.

#### Education

This intervention component was tailored specifically for persons who have undergone a lumbar microdiscectomy. The aim of the educational session is to provide the subjects with baseline knowledge of their past and present back problem, and to discuss strategies to care for their back presently and in the future.

The development of the education program began with determining the format of the educational delivery. A "one-on-one" therapist-to-subject interaction was chosen to closely simulate the current practice pattern and promote an individualized question-and-answer format. A uniquely designed educational booklet will closely guide the one-hour educational session. The educational booklet was developed for the subject to keep.

Initially, we solicited, reviewed, and compared postoperative (microdiscectomy) instructions from the surgeons participating in this study. This information helped to guide the content of the educational booklet and it was the basis for feedback to the surgeons to guide uniformity of post surgical instructions. The booklet was divided into four sections: "Normal Anatomy of The Spine," "Anatomy of Disc Herniation and the Surgery Following It," "Strategies on How to Protect Your Back," and "Commonly Asked Questions."

During the one-hour educational session, the intervention therapist will go through the entire booklet with the subject. A 10-question multiple-choice quiz, which reviews the content of the booklet, was developed and will be administered to the subject at the completion of the educational session. Upon completion of the quiz, the intervention therapist will review the answers with the participant. There will be no specific consequences associated with performance on the quiz.

#### Exercise – USC spine exercise program

##### Trunk strength and endurance program

The trunk strength and endurance program is designed to load the trunk extensors in a graded manner, as determined by a test of their strength and endurance, by varying the angle of the trunk held against gravity. Exercise session intensity will be determined weekly and guided by test results.

**Figure 2:**
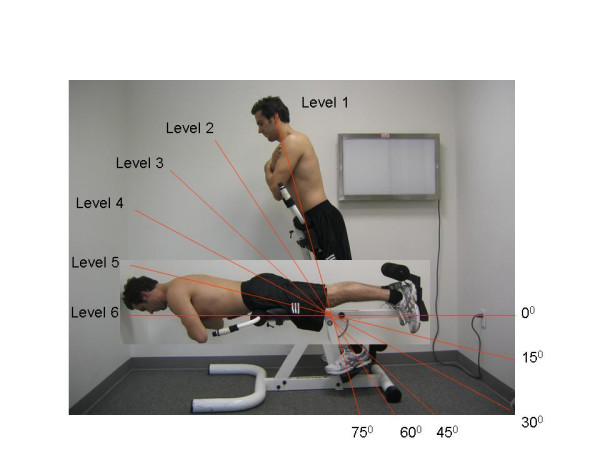
Backstrong Apparatus. Levels of difficulty of the Backstrong apparatus, from easiest (Level 1) to most difficult (Level 6).

There are three main features that distinguish the Strength and Endurance program from other therapeutic interventions that have been used with patients with back pain: it is goal-oriented, performance-based, and periodized. The goal of the program is for subjects to be able to hold the Sorensen test position [23] (i.e., prone/horizontal body position with spine and lower extremity joints in neutral position, arms crossed at the chest, lower extremities and pelvis supported with the upper trunk unsupported against gravity) for 180 seconds. The target hold time was chosen because it exemplified the performance of healthy adults on the Sorensen test [24,25]. There are two implied motor attributes associated with this goal. Subjects must have enough isometric back/hip extensor strength to be able to attain the Sorensen position, and enough isometric endurance to hold that position for 180 seconds. To accomplish this goal, the training program makes exclusive use of the Backstrong Spinal Rehabilitation apparatus (Backstrong LLC, Brea, CA) which, when used in conjunction with a weighted vest, allows the resistance to be progressed from very light to much heavier than the actual Sorensen test. This apparatus allows the angle of a mobile frame to vary from 75° to 0° relative to the horizontal in six increments of increasing difficulty: 75°, 60°, 45°, 30°, 15°, and 0° (Figure 2). Each angle was replaced with a level identifier (1, 2, 3, 4, 5, and 6, respectively) with Level 6 being the position of the original Sorensen test. Training on the Backstrong will be conducted on three non-consecutive days per week.

The program (Table 1) is performance-based in that subjects are to be tested weekly as part of the training, and the next week's program is determined from the previous week's test. Testing will be conducted using the procedures outlined by Flanagan and Kulig [26]. The maximum level attained during each testing session will be used to determine the training level for the following two training days. During the first half of the program, subjects will train at two levels below their maximum tested level. During the second half, they will train at one level below their maximum tested level. This procedure will allow subjects to train at an intensity that is appropriate for each individual based on their performance each week.

The program is periodized such that, after an initial two-week learning phase, subjects will alternate between phases that are designed to improve their back/hip extensor strength, and their back/hip extensor endurance, starting with a strengthening phase. The total length of the program is 12 weeks. There is also an advanced program, for those subjects who reach the goal (level 6 for 180 seconds) before the end of the 12 weeks.

During the learning phase, subjects will be taught the proper procedures for mounting and dismounting the exercise equipment, correct positioning, and testing procedures. This phase will end with a maximal test that correctly identifies the training level to begin the first strength phase.

There will be a maximum of two strength phases, each lasting a maximum of two weeks, and there will be two endurance phases, each lasting three weeks. The strength phases alternate with the endurance phases, beginning with the first strength phase. The goal of the strength phases is for the subjects to maintain the position of the original Sorensen test for at least 20 seconds. If subjects achieve this goal by the end of the first strength phase, they will not perform the second strength phase and, instead, will advance to the first endurance phase early and, then, directly to the second endurance phase after the first endurance phase.

The goal of the first endurance phase is for the subjects to be able to hold a sub-maximal position (two levels below their respective individual maximal level) for 90 seconds, while the goal of the second phase is for the subjects to be able to hold the Sorenson position (Level 6) for 180 seconds. Rest periods between repetitions of the isometric contractions will vary depending on the duration of each contraction, and will be calculated using a work-to-rest ratio (as opposed to the fixed 30-second rest in the strength phase). If the work-to-rest ratio is 1:1.5, the rest period will be calculated by multiplying the hold time by 1.5. A similar procedure will be used for work to rest ratios of 1:1 and 1:0.5. Repetitions, sets, and rest periods were designed to provide a safe progression of resistance during the intervention period.

If subjects are advanced early because they reach the particular goal of a phase, they will follow an advanced program for the remainder of the 12 weeks. This program features alternating strength and endurance phases performed at Level 6 with added resistance via a weighted vest (progressing from 5–20% of a patient's body weight). For each subject, the program ends on the 35^th ^training day with the modified Sorensen test, regardless of the point in the program at which the subject might be.

##### Mat and upright program

The purpose of the Mat and Upright Therapeutic Exercise program is to develop sound and individualized strategies to progressively and dynamically develop strength, endurance, and control of movement by the trunk and lower extremity musculature. The systematic and individualized progression and/or regression of exercise intensity is unique to this protocol and mimics the clinical decision-making process employed by physical therapists.

**Figure 3:**
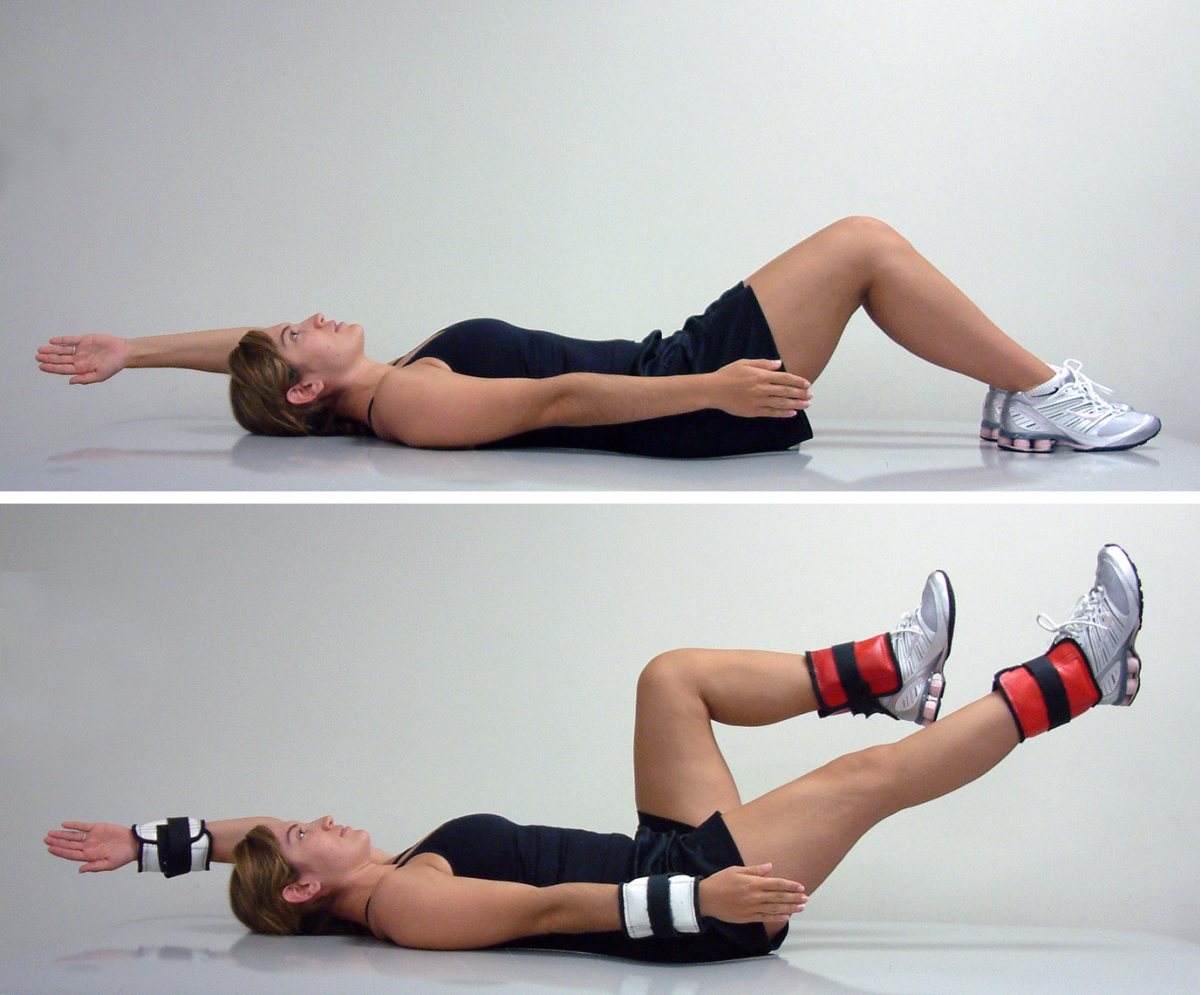
Mat and Upright Therapeutic Exercises. Examples of the easiest (top) and most challenging (bottom) exercises from the "abdominal progression" of the mat and upright program of therapeutic exercises.

**Figure 4:**
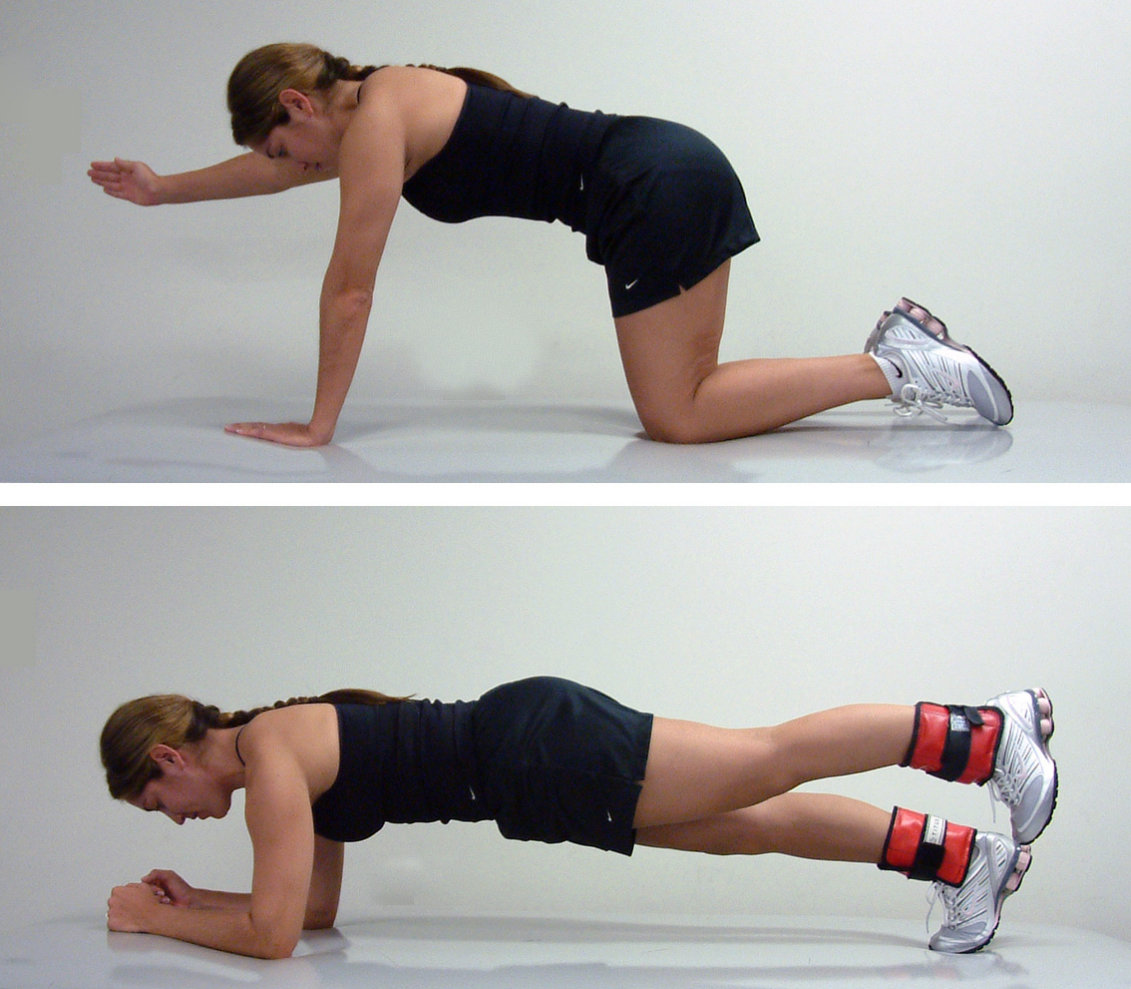
Mat and Upright Therapeutic Exercises. Examples of the easiest (top) and most challenging (bottom) exercises from the "quadruped progression" of the mat and upright program of therapeutic exercises.

**Figure 5:**
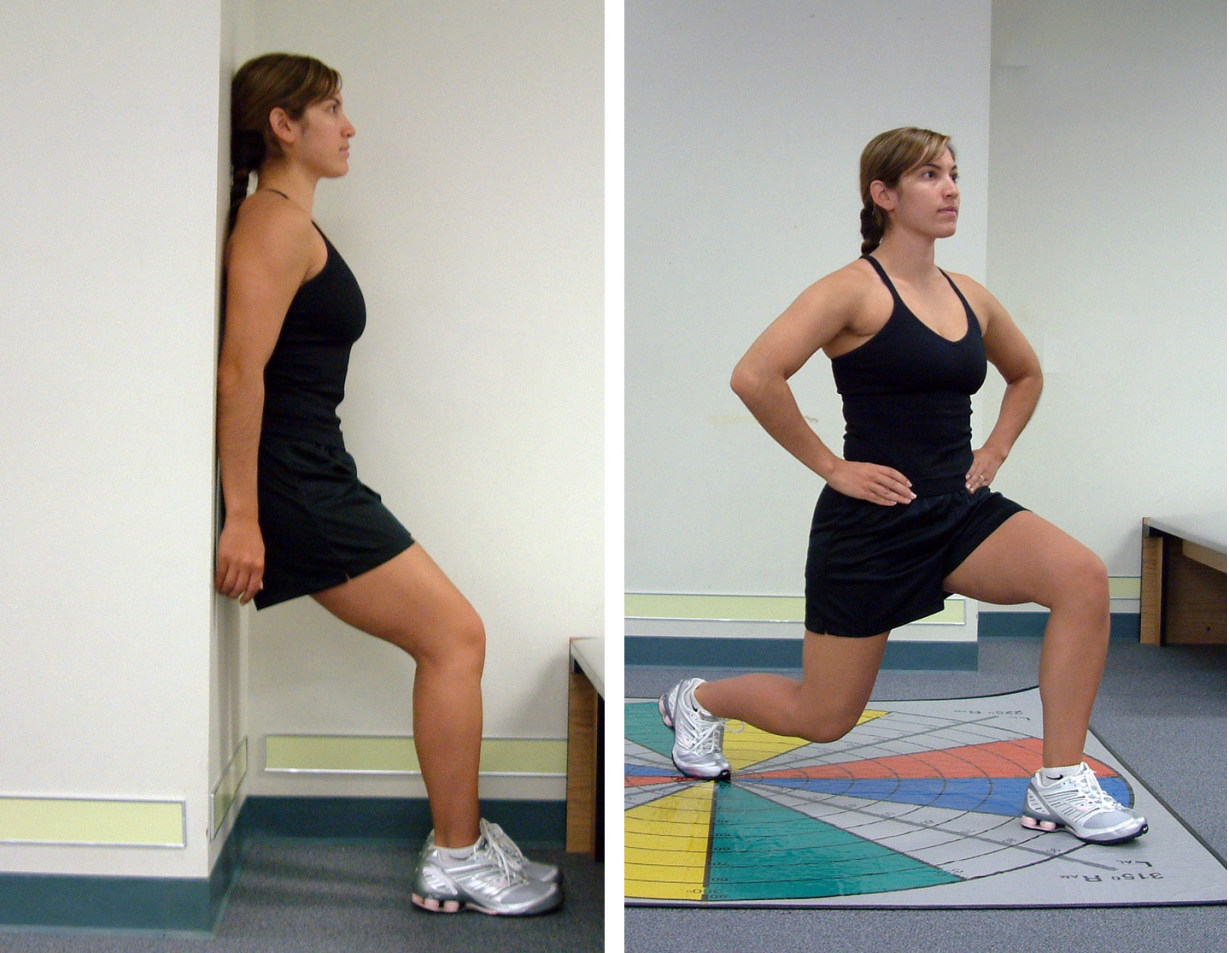
Mat and Upright Therapeutic Exercises. Examples of the easiest (left) and most challenging (right) exercises from the "squat/lunge progression" of the mat and upright program of therapeutic exercises.

Three categories of exercises were selected to focus on the abdominal, back, and lower extremity musculature. A progression of these exercises of increasing difficulty was established (Table 2). The exercises are divided into three categories based on performance in supine, quadruped, and standing positions. Examples of the easiest and most challenging exercises are presented in Figures 3, 4, and 5, respectively. The exercise selection was guided by literature review [27-29], clinician expertise input via focus group discussions, and field testing. Repetitions, sets, and rest periods were further determined based on muscle endurance training principles set by the American College of Sports Medicine [30]. Each category of exercise has multiple training levels designed to accommodate subjects of varying levels of fitness and symptoms, and to allow for progression of the workload over the 12-week training period. Subjects will perform exercises from all three categories during the entire intervention period, but the level of difficulty of exercises at any point during the period may vary among the exercise categories.

A testing procedure was developed to determine the appropriate initial training level for each category of exercise. Test performance will be based upon each subject's symptoms, technique, and rate of perceived exertion. The tests will be repeated at three-week intervals during the 12-week intervention. Results will be used for data collection and to modify training levels.

Exercise performance was also evaluated to allow for progression, regression, or maintenance of exercise within each category on a weekly basis. Once a training goal is met two sessions in a row, the subject will progress to the next training level. If a subject fails to complete one-half of the target reps, hold times, or sets, or is symptom-limited for two consecutive sessions, the subject will be regressed by one level. This allows for an individualized program based on symptoms, fatigue, and ability to perform the exercise correctly.

### Outcome measures

**Figure 6:**
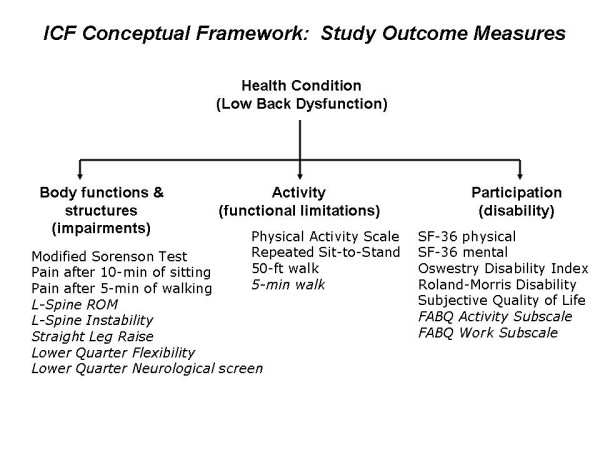
Study outcome measures categorized under the ICF framework, with corresponding Nagi framework categories shown in parentheses. The secondary outcome measures are shown in italics.

We have used the International Classification of Functioning (ICF) conceptual framework [31,32] to classify the study's outcome measures (Figure 6). The ICF framework categories (body functions and structures, activity, participation) correspond, respectively, to Nagi's disablement model [32,33] categories (impairments, functional limitation, disability). We divided the outcome measures into primary and secondary variables. The primary outcome measures will provide an assessment of the intervention and the secondary outcome measures are descriptive, informing, and hypothesis-building. Testing of all outcome measures will be administered at the post-surgical/pre-intervention assessment, post-intervention assessment, and 1-year post-surgical assessment. In addition, completion of questionnaires and pain visual analog scales (VASs) will occur every six months after the 1-year post-surgical assessment, for the remaining four years of the study.

The subject must complete all sections of each questionnaire for the questionnaire to be considered valid. Research personnel will ensure that all sections are completed during each assessment. Incomplete questionnaires will not be accepted for data entry.

#### Primary outcome measures

The following survey instruments were placed in the "participation" category of the ICF: Oswestry Disability Questionnaire (OD), Roland-Morris Disability Questionnaire (RM), SF-36^® ^quality of life assessment, and Subjective Quality of Life Scale (SQOL).

The OD measures the activities of daily living most likely to be limited in people with lower back pain. The most recent version of the OD will be used, in which the question regarding sex has been replaced with one regarding employment and homemaking [34]. The RM is a short questionnaire that assesses functional intolerances related to low back pain. It is easy to administer and has been shown to be reliable [35,36] Both the OD and the RM have been used as outcome measures with patients who have undergone microdiscectomy [7,9-11,37-39].

The SF-36^® ^has been used with a variety of populations to compare the effects of different diseases and treatments, and for screening individuals' health profiles [40]. For the purposes of this study, the SF-36^® ^will be used to assess baseline quality of life for each subject before and after the intervention period. It has been used in patients with low back pain and after lumbar surgeries [37]. The SQOL [41] comprises only one item, and is used to obtain a general quality of life assessment.

The "activity" primary outcome measures will include the 50-foot Walk and Repeated Sit-to-Stand tests. The 50-foot Walk test measures the time it takes to walk a distance of 50 feet as fast as tolerated. The Repeated Sit-to-Stand test measures the time it takes to complete five consecutive repetitions of a sit-to-stand sequence as fast as tolerated. Good-to-excellent test-retest reliability has been established for these measures (ICC = 0.99 and 0.89, respectively) [42].

The "body functions and structures" (i.e., "physical") primary outcome measures will include a Modified Sorenson test and pain visual analog scales [43] (VASs). The pain VASs will be administered immediately after sitting for 10 minutes and after the 5-minute Walk test.

The Modified Sorensen test was derived from Biering-Sorensen [23], who used this test to assess the isometric strength and endurance of the lumbar back extensors in individuals with low back pain. The purpose of the Modified Sorensen test in our study is to assess the isometric strength and endurance of the lumbar musculature of each subject. The Sorensen test was shown to be reliable when used to test subjects who had a history of current or previous episodes of lower back pain [24,42]. In addition, the test was determined to be clinically useful because it was easy to perform, required minimal equipment, and had the most support in the literature [19,24,42,44]. Finally, the Sorensen test was found not to be linked to genetic attributes of strength, and therefore is more suitable for a rehabilitative outcome measure [45].

#### Secondary outcome measures

The Fear Avoidance Belief Questionnaire (FABQ) is a secondary "participation" outcome measure in our study. It focuses specifically on a subject's belief about how physical activity and work affects low back pain [46]. Fritz et al [47] determined that screening for fear-avoidance beliefs may be useful for identifying subjects at risk of prolonged disability and work absence.

Secondary "activity" outcome measures include the 24-hour Physical Activity Scale (PAS) and the 5-minute walk test. The PAS is a survey that requires the subjects to estimate the amount of time spent at different types and levels of physical activity during a typical day [48]. Its purpose is to monitor the level of activity in the subjects enrolled in the study, both as an outcome measure as well as an activity-monitoring strategy for all subjects. The 5-minute walk test will measure how far each subject can walk in a 5-minute period. The speed is self-selected by the subject. Excellent test-retest reliability (ICC = 0.99) has been established for this measure [42].

Secondary "physical" outcome measures include the following tests: lower quarter neurological screen, straight leg raising (SLR), lower quarter flexibility, lumbar spine range of motion, and lumbar spine instability. The purpose of the lower quarter neurological screen is to assess sensation, strength, and deep tendon reflexes (DTRs) before and after the intervention. The screen was derived from several different sources. The sensory testing points were derived and modified from the American Spinal Injury Association (ASIA) screen [49,50]. Sensation to pin prick (sharp/dull discrimination), light touch, and vibration (using a 256-Hz tuning fork) will be assessed. The muscles chosen for manual muscle testing were also derived from the ASIA screen. The muscle testing procedure was derived from Kendall et al [50]. Gastrocnemius/Soleus (S1) manual muscle test grading was based on the work of Lunsford and Perry [51]. Patellar and Achilles DTRs were assessed.

The Passive Straight Leg Raise Test (PSLR) will be used to assess the mechanical movement of the nerves in the lower extremity and to assess symptoms [52,53]. The PSLR will be quantified using a goniometer to measure hip flexion ROM, and a pain VAS to quantify pain complaint. In addition, a crossed straight leg raise (CSLR) sign, in which elevation of the leg produces symptoms on the contralateral spine and/or leg [54], will also be noted. The PSLR test has been shown to have high sensitivity (i.e., a negative PSLR is highly indicative of no presence of a herniated lumbar disc) and low specificity (i.e., a positive test is not highly indicative of the presence of a herniated disc). On the other hand the CSLR sign has been show to have a low sensitivity (0.29) and high specificity (0.88) [54]. A positive PSLR will be considered one in which a patient's symptoms are reproduced by ankle dorsiflexion.

Lower quarter flexibility/muscle length measurements will be assessed to determine the effect of the intervention on muscle flexibility, and will be derived from the amount of movement attained at the relevant joint for each muscle group. Lumbar spine range of motion (ROM) will be used to quantify lumbar spine mobility during forward flexion, extension, and right and left lateral flexion for each subject. Due to post-surgical restrictions, lumbar ROM will only be measured immediately following the intervention and one year after surgery. Post-surgical restrictions are in place to prevent the patient's mobility from compromising the surgery. Lumbar spine ROM in the sagittal plane will be assessed using a double inclinometer method, which has been proven to be a highly reliable and valid measurement technique [55]. Lumbar spine ROM in the frontal plane will be assessed using a tape measure.

Lumbar spine instability will be tested to gain information on the nature of the subject's symptoms. With the subject's torso prone on a treatment table and both lower extremities on the floor, the examiner will apply posterior-to-anterior (P-A) pressure at each lumbar spinous process to the end of the perceived range of motion. Reproduction of pain, and the involved segment, will be noted. If the subject reports a full reduction of symptoms at the painful segment when lifting the LEs, it will be considered a positive test for instability of that segment. The prone instability test has been shown to be more reliable than the segmental instability test [56].

### Follow-up period

All of the previously described questionnaires will be administered during face-to-face evaluation sessions occurring four to six weeks after surgery, at the end of the intervention period, and one year after the surgery. They will be administered via the regular mail during the follow-up period (every six months thereafter for the next four years). The mailings will be preceded by phone calls from the study coordinators during which the subjects will be asked questions regarding their symptoms and activity level. If the subjects report that they are symptomatic, then the following questionnaires will be sent: OD, RM, SF-36^®^, SQOL, FABQ, and PAS. In this case, subjects will also receive the pain VAS forms for sitting and walking, and will be asked to report their typical symptoms (intensity and location) during these activities, but they will not need to mark the pain VAS forms immediately after sitting or walking for a certain time. If, however, the subjects report that they are asymptomatic at the time of the follow-up call, only the PAS will be sent.

### Data analysis

The primary and secondary outcome measures will be assessed for immediate and long-term effects using two-factor multivariate analysis of variance (MANOVA), with repeated measures for time. For all multivariate ANOVA tests described, significant main effects will be reported if there are no significant interactions. If significant interactions are found, then one-way ANOVAs will be performed for each main effect. Post-hoc testing will be performed (as necessary) to determine statistically significant pairwise comparisons. Statistical analysis of the results will be performed using the SAS system, version 8.2 (SAS Institute Inc., Cary, NC, USA). All significance levels will be set at p < 0.05.

### Sample size

Power calculations were based on data from prior published studies [23,24,42,44,57]. These data indicated that the inter-subject variability with respect to impairments, activity, and participation, was moderate to high. Since the outcome measures assess the immediate and long-term effects of rehabilitation in addition to surgery, the calculations were made on the assumption that the changes due to exercise and education will be greater than those expected for education alone. A 50% difference between groups (education, and exercise plus education) is expected based on prior studies, and a 30% subject attrition rate is anticipated. The possible attrition rate is attributed to length of the rehabilitative program, elimination of symptoms, or increase of symptoms and need for a second surgery. All power calculations were based on an alpha level of 0.05 and power of 0.8. Therefore, accounting for attrition, each group would need approximately 50 subjects.

## Discussion

We have presented the design and rationale for an RCT assessing the immediate and long-term effects of an individualized, multi-factorial exercise program on body functions and structures, activity, participation, and quality-of-life outcome measures in patients who have undergone a single-level lumbar microdiscectomy for the first time. The results of this trial will be presented as soon as they become available.

## Competing interests

The author(s) declare that they have no competing interests.

## Authors' contributions

SPF, KK, NDM, EMP and CMP were responsible for the design of this study. KAY is the study's coordinator and WB is the study's recruiter. DS is a study evaluator and prepared the current manuscript. GJB, JRL and JMP are study evaluators. CE provided statistical consultation. All authors read and approved the final manuscript.

## Pre-publication history

The pre-publication history for this paper can be accessed here:



## Figures and Tables

**Table 1:** Trunk Strength and Endurance Program using the Backstrong Apparatus

**Phase**	**Goals**	**Week**	**Training Level**	**Sets**	**Reps**	**Hold Time**	**Rest Length/Reps**	**Rest Length/Sets**
Teaching	1. Correct Technique 2. Identification of Starting Training Level	1	2 Levels < Submax Test Level	1	4	30	30	NA
		2	2 Levels < Submax Test Level	1	4	30	30	NA
Strength I	1. Level 6 for 20 seconds	3	2 Levels < Max Test Level	2	3	30	30	60
		4	2 Levels < Max Test Level	3	3	30	30	60
Endurance I	1. Submax level for 90 seconds	5	2 Levels < Max Test Level	1	6–8	Max*	Max*	NA
		6	2 Levels < Max Test Level	1	8–10	Max*	Max*	NA
		7	2 Levels < Max Test Level	1	8–10	Max*	Max*	NA
Strength II	1. Level 6 for 20 seconds	8	1 Level < Max Test Level	4	5	30	30	60
		9	1 Level < Max Test Level	5	5	30	30	60
Endurance II	1. Level 6 for 180 seconds	10	1 Level < Max Test Level	2	4	Max*	Max*	180
		11	1 Level < Max Test Level	2	5	Max*	Max*	180
		12	1 Level < Max Test Level	2	6	Max*	Max*	180

* Up to 90 seconds

**Table 2:** Mat and Upright Therapeutic Exercise Program (see Figures 3, 4 and 5)

**EXERCISE**	**TRAINING GOAL**
**Abdominal Progression**	

**Level 1**	3 sets of one minute continuous motion
Supine Alternating (Alt) UE Flexion	1 minute of rest between sets
**Level 2**	3 sets of one minute continuous motion
Supine Alt LE Extension	1 minute of rest between sets
**Level 3**	3 sets of one minute continuous motion
Supine Alt UE Flexion & LE Extension	1 minute of rest between sets
**Level 4**	3 sets of two minute continuous motion
Supine Leg Ext Unsupported	2 minute of rest between sets
**Level 5**	3 sets of two minute continuous motion
Supine Leg Ext Unsupported w/Alt Arms	2 minute of rest between sets
**Level 6**	3 sets of two minute continuous motion
With 1# and 3# Weights	2 minute of rest between sets
**Level 7**	3 sets of two minute continuous motion
With 2# and 5# Weights	2 minute of rest between sets

**Quadruped Progression**	

**Level 1**	10 repetitions with 10 second hold per extremity raise
Alt Arm Raises	No resting time
**Level 2**	10 repetitions with 10 second hold per extremity raise
Alt Leg Ext	No resting time
**Level 3**	10 repetitions with 10 second hold per extremity raise
Alt Arm and Leg Raises	No resting time
**Level 4**	6 repetitions with a 30 second hold per repetition
Prone Plank on Knees	30 seconds rest between repetitions
**Level 5**	6 repetitions with a 30 second hold per repetition
Prone Plank on Forefoot	30 seconds rest between repetitions
**Level 6**	6 repetitions with a 15 second hold per leg raise per repetition
Prone Plank w/alt Leg Lift	30 seconds rest between repetitions
**Level 7**	6 repetitions with a 15 second hold per leg raise per repetition
Prone Plank w/alt Leg Lift w/3#	30 seconds rest between repetitions
**Level 8**	6 repetitions with a 15 second hold per leg raise per repetition
Prone Plank w/alt Leg Lift w/5#	30 seconds rest between repetitions

**Squat/Lunge Progression**	

**Level 1**	3 sets of 20 repetitions
Wall Squat to 45° Knee Flexion	5 second hold per repetition. 2 minutes rest between sets
**Level 2**	3 sets of 20 repetitions
Free Standing Squats to 90° Hip Flexion	2 minute of rest between sets
**Level 3**	3 sets of 20 repetitions
Forward Lunges	2 minute of rest between sets
**Level 4**	3 sets of 2 cycles
Lunges Series	2 minute of rest between sets
**Level 5**	3 sets of 3 cycles
Lunge Series	2 minute of rest between sets
